# Ambulatory Blood Pressure Phenotypes, Arterial Stiffness, and Cardiac Remodeling

**DOI:** 10.1093/ajh/hpae106

**Published:** 2024-08-08

**Authors:** Cesare Cuspidi, Rita Facchetti, Elisa Gherbesi, Fosca Quarti-Trevano, Jennifer Vanoli, Giuseppe Mancia, Guido Grassi

**Affiliations:** Department of Medicine and Surgery, University of Milano-Bicocca, Milano, Italy; Department of Medicine and Surgery, University of Milano-Bicocca, Milano, Italy; Department of Cardio-Thoracic-Vascular Diseases, Foundation IRCCS Ca’ Granda Ospedale Maggiore Policlinico, Milano, Italy; Department of Medicine and Surgery, University of Milano-Bicocca, Milano, Italy; Department of Medicine and Surgery, University of Milano-Bicocca, Milano, Italy; Department of Medicine and Surgery, University of Milano-Bicocca, Milano, Italy; Department of Medicine and Surgery, University of Milano-Bicocca, Milano, Italy

**Keywords:** arterial stiffness, blood pressure, CAVI, hypertension, left ventricular hypertrophy, left ventricular remodeling, sustained hypertension

## Abstract

**BACKGROUND:**

Evidence on the association of arterial stiffness and left ventricular (LV) concentric remodelling/LVH assessed by echocardiography, with abnormal blood pressure (BP) phenotypes, defined by office and ambulatory BP monitoring (ABPM) in the community is scanty. Thus, we investigated this issue in the participants to the Pressioni Monitorate E Loro Associazioni (PAMELA) study.

**METHODS:**

The present study included 491 participants who attended the second and third survey of the PAMELA study performed after 10 and 25 years from the initial evaluation. Data collection included medical history, anthropometric parameters, blood examinations, office, ABPM, echocardiographic and Cardio-Ankle Vascular Index (CAVI) measurements.

**RESULTS:**

In the whole study sample (age 66+10 years, 50% males), the prevalence rates of sustained normotension (NT), white coat hypertension (WCH), masked hypertension (MH), sustained hypertension (SH) and non-dipping (ND) were 31.2, 10.0, 24.2, 34.6, and 35.8% and respectively. The likelihood of having SH, the BP phenotype carrying the greatest CV risk, was four times higher (OR= 4.31, CI:2.39-7.76, p<0.0001) in participants with increased CAVI and LV remodelling/LVH compared to their counterparts without organ damage. This association showed an incremental value in discriminating SH compared to both isolated markers of organ damage (OR=1.92,p=0.03 for increased CAVI and OR= 2.02, p=0.02 for LV remodelling/LVH). The presence of isolated but also combined organ damage was unrelated to ND.

**CONCLUSIONS:**

Our study provides new evidence of the incremental value of looking for both vascular and cardiac target organ damage to optimize the identification and clinical management of SH in the general population.

## Graphical Abstract

**Graphical Abstract:**
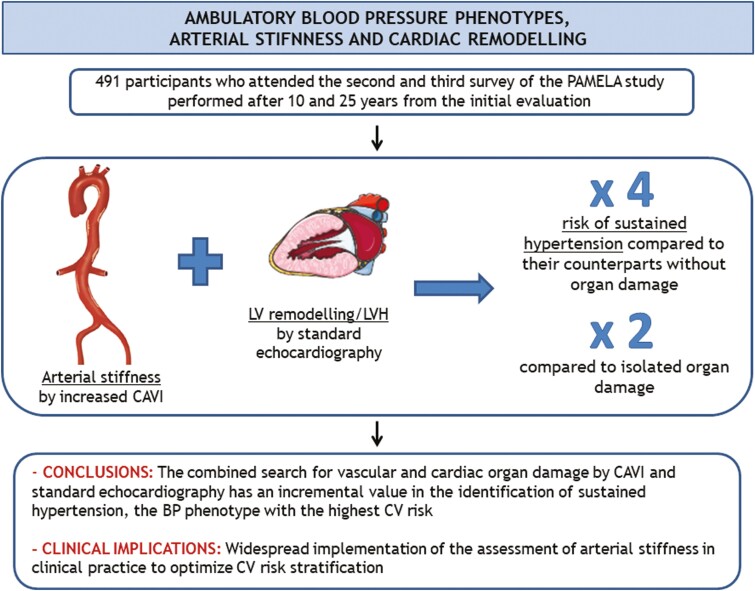

Several lines of evidence have shown that blood pressure (BP) measured outside the medical environment is more closely associated with hypertension-mediated organ damage (HMOD), which is universally recognized as an intermediate step in the disease continuum linking risk factors to cardiovascular (CV) events and all-cause mortality.^1–3^

In the last 4 decades, out-of-office (i.e., ambulatory or home) BP measurements have increasingly been adopted in clinical practice allowing for a more precise evaluation of the patient’s BP status and consequently of the inherent CV risk.^4–6^ By this well-established approach, 4 different BP phenotypes may be identified, namely true normotension (NT) (i.e., normal office and out-of-office BP), white coat hypertension (WCH) (i.e., elevated office and normal out-of-office BP), the opposite pattern known as masked hypertension (MH) (i.e., normal office and elevated out-of-office BP), and sustained hypertension (SH) (i.e., elevated in-office and out-of-office BP).^7,8^

Furthermore, out-of-office BP measured by the 24-hour ambulatory BP monitoring (ABPM) has the unique capability to assess day-night BP changes allowing to classify individuals as dippers (i.e., with a fall in nighttime BP >10% compared to daytime BP values) and non-dippers (i.e., with a <10% BP reduction of nighttime vs. daytime BP values).^9,10^

CV risk stratification based on office and out-of-office BP implemented by the search for HMOD has important implications in clinical and therapeutic decisions.^6,11^ Although a large body of evidence has accumulated on the association between abnormal BP phenotypes and various markers of HMOD, very few studies focused both on arterial stiffness and cardiac remodeling, phenotyped by cardio-ankle vascular index (CAVI) and echocardiography, in general, population-based samples.^12^

To add a new piece of evidence in this research field, we assessed the relationship of CAVI and left ventricular (LV) remodeling/LV hypertrophy (LVH) with BP phenotypes among the participants to the third survey of Pressioni Arteriose Monitorate e Loro Associazioni (PAMELA) study in which clinical, laboratory, ABPM, echocardiographic, and arterial stiffness measurements were prospectively collected.^13^ Thus, we tested the hypothesis that a combined search for subclinical vascular and cardiac organ damage may have additional value in identifying high-risk BP phenotypes (i.e., SH and non-dipping [ND]) compared to the isolated evaluation of vascular or cardiac HMOD.

## METHODS

As described in detail elsewhere,^14^ at the initial evaluation, carried out between 1990 and 1993, a total of 2,051 participants to the PAMELA Study, representative of the population of Monza (a city of over 1,000 inhabitants near Milan, Italy) stratified according to sex, age (decades) from 25 to 74 years of age were examined. After an informed consent, participants were invited to the outpatient clinic of the S. Gerardo Hospital of Monza for a comprehensive clinical, laboratory, and instrumental evaluation. Data collection included a medical history, weight, height, abdominal circumference, standard blood exams, office, home, and 24-hour ABPM, transthoracic echocardiography.

Office BP was measured three times with the subject in the sitting position, using a mercury sphygmomanometer in the first and second surveys and an oscillometric device in the third survey (Takeda TM-2430 A&D). To measure ABP, participants were fitted with an ABPM device (Spacelabs 90207, Issaquah, WA, United States) set to obtain automated oscillometric BP and heart rate readings over a 24-hour period. Participants were also asked to self-measure BP at home, with a validated semiautomatic oscillometric device (model HP 5331, Philips), at approximately 7 pm and 7 am, using the arm contralateral to the one used for ABPM. Two home measurements were taken on each occasion at a 1-minute intervals.

Participants were again contacted from 2001 to 2003, i.e., after a mean time interval of 10.7 ± 0.61 years, and from 2017 to 2018, i.e., after a mean time interval of 25.4 ± 1.0 years, and those willing to be re-examined were asked to attend the San Gerardo University Hospital for a second and third set of data collection, according to the same procedures used for the first set of data collection. Furthermore, unlike the previous two evaluations, the participants in the third survey underwent CAVI measurement.

### Ambulatory blood pressure monitoring

Subjects were then fitted with an ABPM device set to obtain automated BP and heart rate oscillometric readings every 20 minutes over 24 hours. The subjects were asked to pursue their normal activities during the monitoring period with the precaution of holding the arm still at the time of BP reading, of going to bed no later than 11.00 pm, and arising not before 7.00 am. All ABPMs were performed on a working day (Monday–Friday). Recordings were analyzed to obtain 24-hour, daytime (7.00 am to 11.00 pm) and nighttime (11.00 pm to 7.00 am) average systolic BP (SBP)/diastolic BP (DBP), nocturnal SBP/DBP percent decrease, and heart rate.

### Definition of BP phenotypes

Participants with normal office BP values (<140/90 mm Hg,) were categorized into NT and MH groups, based on the normality or elevation of 24-hour mean BP set by the hypertension guidelines,^6,11^ i.e., < or ≥ than 130 mm Hg systolic and/or 80 mm Hg diastolic BP, respectively. Participants with elevated office BP values (≥140/90 mm Hg,) were categorized into WCH and SH groups based on the normality or elevation of 24-hour mean BP. Nocturnal ND pattern was defined as a nighttime reduction in SBP lower than 10% compared to daytime values.

### Echocardiography

Transthoracic echocardiographic data were collected according to standard procedures, as previously reported.^15^ In brief, M-mode and 2-dimensional echo examinations were carried out with a commercially available instrument (Acuson 128 CF, Computer Sonography, and Samsung Medison EKO 7). End-diastolic (d) and end-systolic (s) LV internal diameters (LVID), interventricular septum (IVS) thickness, and posterior wall (PW) thickness were measured off-line from 2-dimensionally guided M-mode tracings recorded at 50–100 cm/s speed, during at least 3 consecutive cycles. LV mass was estimated by using the corrected ASE method: 0.8 × (1.04 × [(IVSd + LVIDd + PWTd)^3^ − LVIDd^3^]) + 0.6 and normalized to body surface area (BSA).^16^ Relative wall thickness (RWT) was calculated as the ratio of PW plus IVS thickness to LVIDd. LVH was defined as LV mass (LVM) index (LVMI) higher than 115 g/m^2^ in men and 95 g/m^2^ in women. LV concentric remodeling was defined as normal LVMI and increased RWT (i.e., ≥0.43).^6^

### Cardio-ankle vascular index

The CAVI was measured and automatically calculated using the VaSera system (Fukuda Denshi Co, Japan). Measurements were performed in a quiet room at a stable temperature of 22–24 °C with fasting patients, advised to avoid physical activity and smoking at least 1 hour before, immediately following the echocardiographic examination.

According to the manufacturer’s recommendations, electrocardiogram electrodes were placed on both wrists, a microphone for phonocardiography on the sternum, and 4 BP cuffs wrapped around the 4 limbs. The upper arm and ankle pulse waves, as well as BP, were measured. The intra- and inter-observer coefficients of variation for CAVI were 1.9% and 2.2%, respectively. Increased arterial stiffness was defined by a threshold value of CAVI >9.0 m/s which represents the value above the median obtained in the PAMELA population in line with the abnormal cut-off proposed for the definition of high-risk patients by the Japanese Society for Vascular Failure.^17^

### Statistical analysis

In each subject, the 3 clinic BP values were averaged; also 24-hour ambulatory BP values were averaged after artefactual readings had been eliminated according to preselected criteria.^18^ Valid ambulatory systolic and diastolic BP readings were 95.2% and 94.8%, respectively of the planned 72 readings.

Data related to subjects’ characteristics were analyzed by descriptive ANOVA, Kruskal–Wallis test and chi-square test were used to compare the demographic and clinical characteristics of the subjects in different groups. Bonferroni correction was used to compare the two groups. The association between BP phenotypes and subclinical vascular and cardiac organ damage patterns was evaluated by the Chi-square test. The Cochran–Mantel–Haenszel (CMH) method was used to adjust the *P*-value of association by sex and age (under and below the median value).

The association between SH (and ND pattern) and subclinical vascular and cardiac organ damage patterns was also evaluated by logistic models. Odds Ratio (OR) was reported unadjusted and adjusted for age and sex. Subjects with normal stiffness and normal LV geometry and mass were taken as references.

A *P*-value <0.05 was considered statistically significant. Statistical analysis was performed by SAS System (version 9.4; SAS Institute Inc, Cary, NC, United States).

## RESULTS

From the original sample of 2,051 individuals who underwent the initial examination, 1,412 participated in the second survey, and 562 attended the third survey. Echocardiographic, CAVI, and ABPM data were available in 491 of them.

The clinical features of these participants are summarized in Table 1. The mean age was 66 ± 10 years and the prevalence of the male gender was 50.5%. Average body mass index (BMI) and waist circumference (WC) were 26.3 ± 4.2 kg/m^2^ and 91.3 ± 13.2 cm, respectively. Mean office BP was 136 ± 17/83 ± 9 mm Hg, mean home BP 128 ± 16/78 ± 9 mm Hg, and mean 24-hour BP 133 ± 14/78 ± 7 mm Hg with 48% of participants being treated with anti-hypertensive drugs. The prevalence rates of the 4 BP phenotypes according to the criteria reported in the “Methods” section, and the ND pattern were as follows: 31.2% NT, 10.0 % WCH, 24.2% MH, 34.6% SH, and 35.8%. Clinical data regarding participants grouped according to this BP classification are reported in Supplementary Table 1.

**Table 1.** Demographic and clinical characteristics of the participants to the third survey of the PAMELA study

	*N* = 491
Age (y)	66.1 ± 9.5
Male (%)	50.5
Body mass index (kg/m^2^)	26.3 ± 4.2
Waist circumference (cm)	91.3 ± 13.2
Office SBP (mm Hg)	136.5 ± 17.5
Office DBP (mm Hg)	83.1 ± 8.8
Office HR (mm Hg)	70.3 ± 10.1
Home SBP (mm Hg)	127.7 ± 16.3
Home DBP (mm Hg)	77.7 ± 8.9
Home HR (mm Hg)	70.7 ± 9.6
24-h SBP (mm Hg)	133 ± 13.7
24-h DBP (mm Hg)	77.6 ± 7.5
24-h HR (mm Hg)	71.9 ± 7.7
Daytime SBP (mm Hg)	138.1 ± 14.3
Daytime DBP (mm Hg)	81.4 ± 8.1
Daytime HR (mm Hg)	75.2 ± 8.1
Nighttime SBP (mm Hg)	119.9 ± 15.7
Nighttime DBP (mm Hg)	68 ± 8.4
Nighttime HR (mm Hg)	63.2 ± 8
Antihypertentive treat (%)	48.1
Total cholesterol (mg/dl)	200.9 ± 37
HDL cholesterol (mg/dl)	59 ± 17.2
Serum glucose (mg/dl)	90 (84–100)
Triglycerides (mg/dl)	96 (73–127)
Uric acid (mg/dl)	5 ± 1.3
Serum creatinine (mg/dl)	0.93 ± 0.23
LVM/BSA (g/m^2^)	85.5 ± 20.3
CAVI (m/s)	9.2 ± 2.1
Sustained normotension (%)	31.2
White coat hypertension (%)	10.0
Masked hypertension (%)	24.2
Sustained hypertension (%)	34.6
Dipper (%)	64.2

Data are shown as means ± SD, median (Q1–Q3), percentages.

Abbreviations: BSA, body surface area; CAVI, Cardio-ankle vascular index; DBP, diastolic blood pressure; HDL, high-density lipoproteins; HR, heart rate; LVM, left ventricular mass; SBP, systolic blood pressure.

Table 2 shows demographic and clinical characteristics of participants categorized according to subclinical vascular and cardiac target organ damage: (I) normal arterial stiffness and LV mass/geometry (34.1%); (II) normal arterial stiffness and LV concentric remodeling/LVH (19.3%); (III) increased arterial stiffness and normal LV mass/geometry (17.3%); and (IV) increased arterial stiffness and LV concentric remodeling/LVH (29.3%). Participants classified in group I (i.e., without subclinical cardiovascular damage) were younger and less frequently men, exhibited lower office, home, 24-hour, daytime, and nighttime SBP, serum glucose, creatinine, uric acid, triglyceride levels, and were less frequently taking anti-hypertensive drugs than those belonging to group IV (i.e., with both vascular and cardiac damage). The opposite trend was observed for cholesterol (total and HDL), triglycerides, and uric acid. Clinical characteristics of participants categorized in group II (i.e., isolated subclinical cardiac damage) were no different from those classified in group III (i.e., isolated vascular damage) with the exception of BMI, which was significantly higher.

**Table 2.** Demographic and clinical characteristics of the participants in the third survey of the PAMELA study categorized according to subclinical vascular and cardiac organ damage: (I) normal arterial stiffness and left ventricular mass/geometry; (II) normal arterial stiffness and concentric remodeling/left ventricular hypertrophy; (III) increased arterial stiffness and normal left ventricular mass/geometry; and (IV) increased arterial stiffness and concentric remodeling/left ventricular hypertrophy

	I	II	III	IV	*P*-trend
Number	167	95	85	144	
Age (y)	60.8 ± 8*^,^**^,^***	64.5 ± 9.1***	66.3 ± 7.9***	73.2 ± 7.6	<0.0001
Male (%)	37.1*^,^**^,^***	55.8	55.3	59.7	0.0001
Body mass index (kg/m^2^)	25.8 ± 4.5*	27.6 ± 4.4**	25.5 ± 4	26.7 ± 3.7	0.2797
Waist circumference (cm)	87.9 ± 13.2*^,^***	95 ± 14.3	89.9 ± 12.7	93.7 ± 11.6	0.0014
Office SBP (mm Hg)	128.7 ± 15.6**^,^***	133.2 ± 14.8***	138.8 ± 16***	146.5 ± 17.1	<0.0001
Office DBP (mm Hg)	81.2 ± 8.3**^,^***	82.9 ± 8.5	84.7 ± 9	84.6 ± 9	0.0002
Office HR (mm Hg)	69.2 ± 8.4	70.4 ± 10.8	69.9 ± 10.7	71.6 ± 10.8	0.0558
Home SBP (mm Hg)	120.3 ± 13.3*^,^**^,^***	127.5 ± 14.1***	128 ± 13.7***	136.4 ± 18	<0.0001
Home DBP (mm Hg)	76.1 ± 7.5*	79.3 ± 8.7	78.2 ± 9.3	78.2 ± 10.1	0.0652
Home HR (mm Hg)	70.7 ± 8.7	70.7 ± 10	70.6 ± 10.4	70.7 ± 9.9	0.9919
24-h SBP (mm Hg)	129 ± 11.8***	132.3 ± 12.1***	132.1 ± 13***	138.8 ± 15.2	<0.0001
24-h DBP (mm Hg)	76.6 ± 6.7	78.5 ± 7	78.1 ± 8	78.1 ± 8.3	0.0939
24-h HR (mm Hg)	72.1 ± 7.1	71.8 ± 8.5	72.2 ± 7.5	71.6 ± 7.9	0.5583
Daytime SBP (mm Hg)	134.6 ± 13.1***	137.4 ± 12.8***	137.2 ± 13.9***	143.1 ± 15.5	<0.0001
Daytime DBP (mm Hg)	80.5 ± 7.6	82.3 ± 7.6	82.1 ± 8.7	81.3 ± 8.8	0.4322
Daytime HR (mm Hg)	75.6 ± 7.6	75 ± 9	75.8 ± 7.8	74.6 ± 8.4	0.3592
Nighttime SBP (mm Hg)	114.3 ± 12.6***	118.8 ± 13.8***	118.9 ± 13.9***	127.6 ± 17.9	<0.0001
Nighttime DBP (mm Hg)	66.2 ± 7.3***	68.7 ± 8.2	67.8 ± 8.4	69.6 ± 9.4	0.0008
Nighttime HR (mm Hg)	63.1 ± 7.7	63.5 ± 8.9	62.6 ± 8	63.5 ± 7.9	0.7964
Antihypertensive treat. (%)	31.1*^,^**^,^***	53.7	48.2	63.9	<0.0001
Total cholesterol (mg/dl)	208.5 ± 32.5***	202.1 ± 37.9	202.7 ± 37.9	190.4 ± 38.6	<0.0001
HDL cholesterol (mg/dl)	65.1 ± 18.4*^,^**^,^***	55.1 ± 14.4	57.8 ± 16.2	55.3 ± 16.1	<0.0001
Serum glucose (mg/dl)	87 (81–93)*^,^**^,^***	91 (85–103)	90 (84–104)	94.5 (85–106)	<0.0001
Triglycerides (mg/dl)	83.5 (65–115)*^,^**^,^***	107.5 (80–145)	107 (76–137)	100.5 (78–125)	0.1466
Uric acid (mg/dl)	4.66 ± 1.15*^,^***	5.25 ± 1.24	5.06 ± 1.13	5.28 ± 1.38	<0.0001
Serum creatinine (mg/dl)	0.89 ± 0.21***	0.96 ± 0.24	0.92 ± 0.21	0.97 ± 0.26	0.0079
LVM/BSA (g/m^2^)	75.3 ± 14.5*^,^***	90.5 ± 23.3**	81.3 ± 13.3***	96.5 ± 20.9	<0.0001
CAVI (m/s)	7.9 ± 0.8**^,^***	7.9 ± 1**^,^***	10.5 ± 1.6	10.9 ± 2.1	<0.0001

For abbreviations see Table 1.

*P < 0.05 vs. II;

**P < 0.05 vs. III;

***P < 0.05 vs. IV.

**Figure 1.**
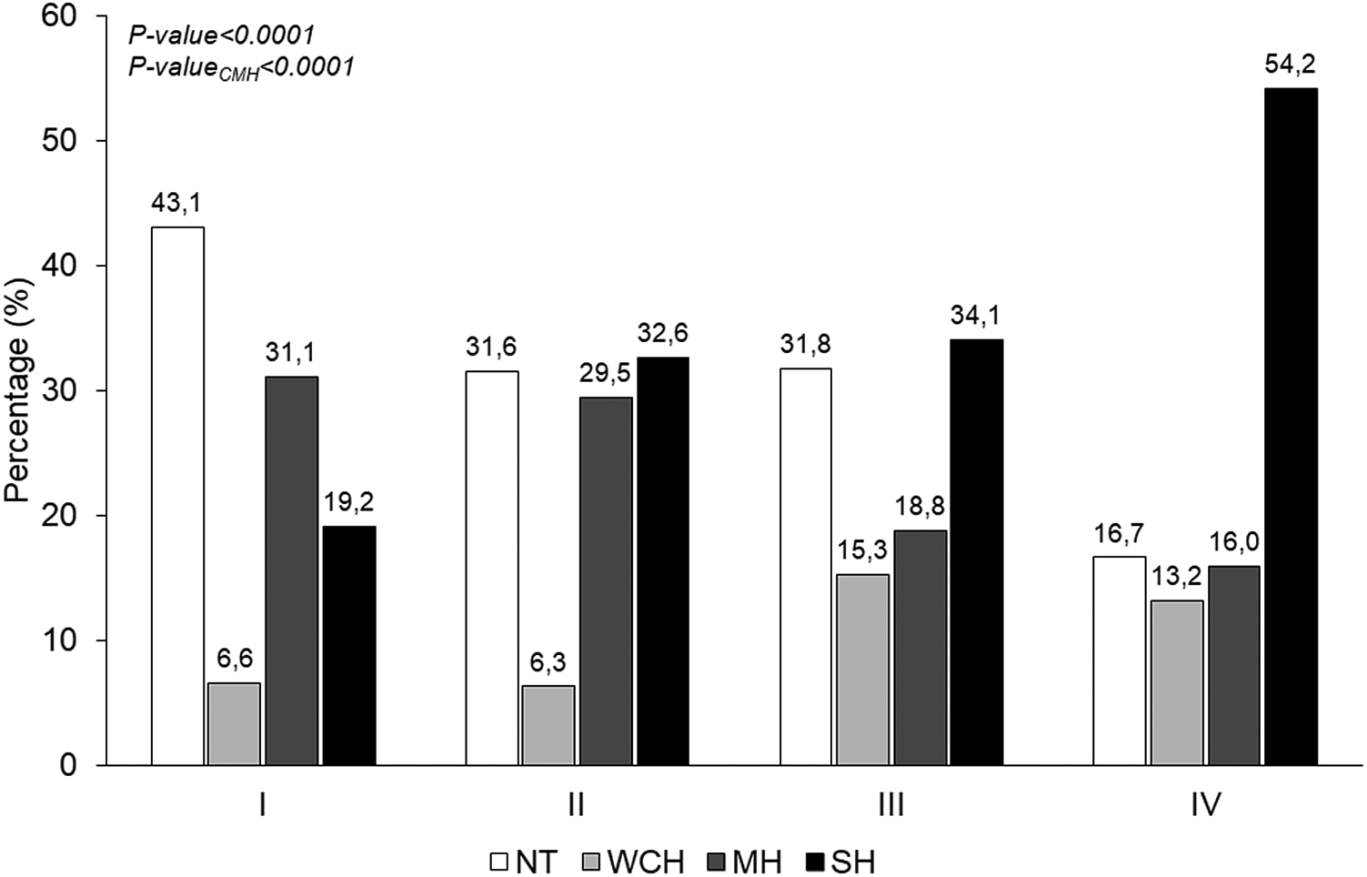
Prevalence rates of blood pressure (BP) phenotypes: normotension (NT), white coat hypertension (WCH), masked hypertension (MH), and sustained hypertension (SH) in the participants to the third survey of the PAMELA study stratified according to hypertension-mediated organ damage (HMOD): (I) normal arterial stiffness, normal left ventricular mass index (LVMI), and geometry; (II) normal arterial stiffness, LV hypertrophy (LVH), or LV concentric remodeling; (III) increased arterial stiffness, normal LVMI, and geometry; and (IV) increased arterial stiffness, LVH, or LV concentric remodeling.

Figure 1 illustrates the prevalence of the NT, WCH, MH, and SH phenotypes across the 4 different categories of vascular and cardiac organ damage. Among the participants with normal arterial stiffness, LV geometry, and LVMI (group I), NT was the most prevalent pattern (43.1%) followed by MH (31.1%), SH (19.1%), and WCH (6.6%); the corresponding values in the group IV were as follows: 16.7%, 16.1%, 54.2%, and 13.2%, respectively. Among the participants with isolated subclinical cardiac damage (group II) and isolated vascular damage (group III), the prevalence of SH was intermediate between groups I and IV, with similar percent values to each other (32.6 % and 34.1%).

**Figure 2.**
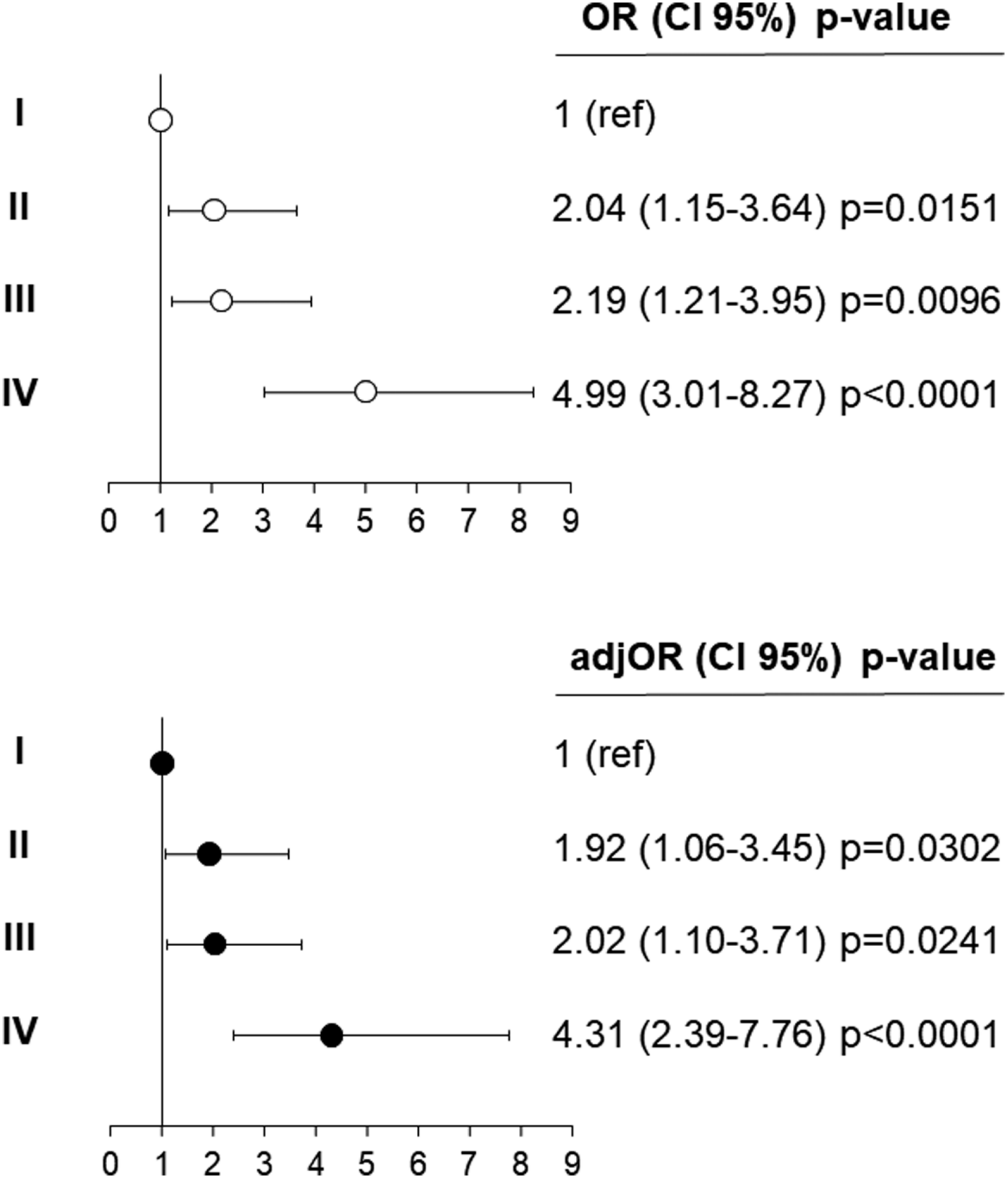
Odds Ratios (ORs), and 95% confidence interval, for the risk of sustained hypertension (SH) in the participants to the third survey of the PAMELA study categorized according to HMOD (II = normal arterial stiffness, LV hypertrophy [LVH], or LV concentric remodeling; III = increased arterial stiffness, normal LVMI and geometry; IV = increased arterial stiffness, LVH, or LV concentric remodeling) having as reference group I (normal arterial stiffness, normal LVMI and geometry). The upper panel and bottom panels show unadjusted and sex/age-adjusted data, respectively. Adj, adjusted.

Figure 2 shows ORs of association with SH (having as reference sustained NT) entailed by the presence of cardiovascular damage in groups II, II, and IV. The likelihood of SH was markedly greater in group IV (OR = 4.31, confidence interval [CI]: 2.39–7.76, *P* < 0.0001) compared to groups II (OR = 1.92, CI: 1.06–3.45, *P* = 0.03) and III (OR = 2.02, CI: 1.10–3.7, *P* = 0.02) after adjusting for age and sex.

**Figure 3.**
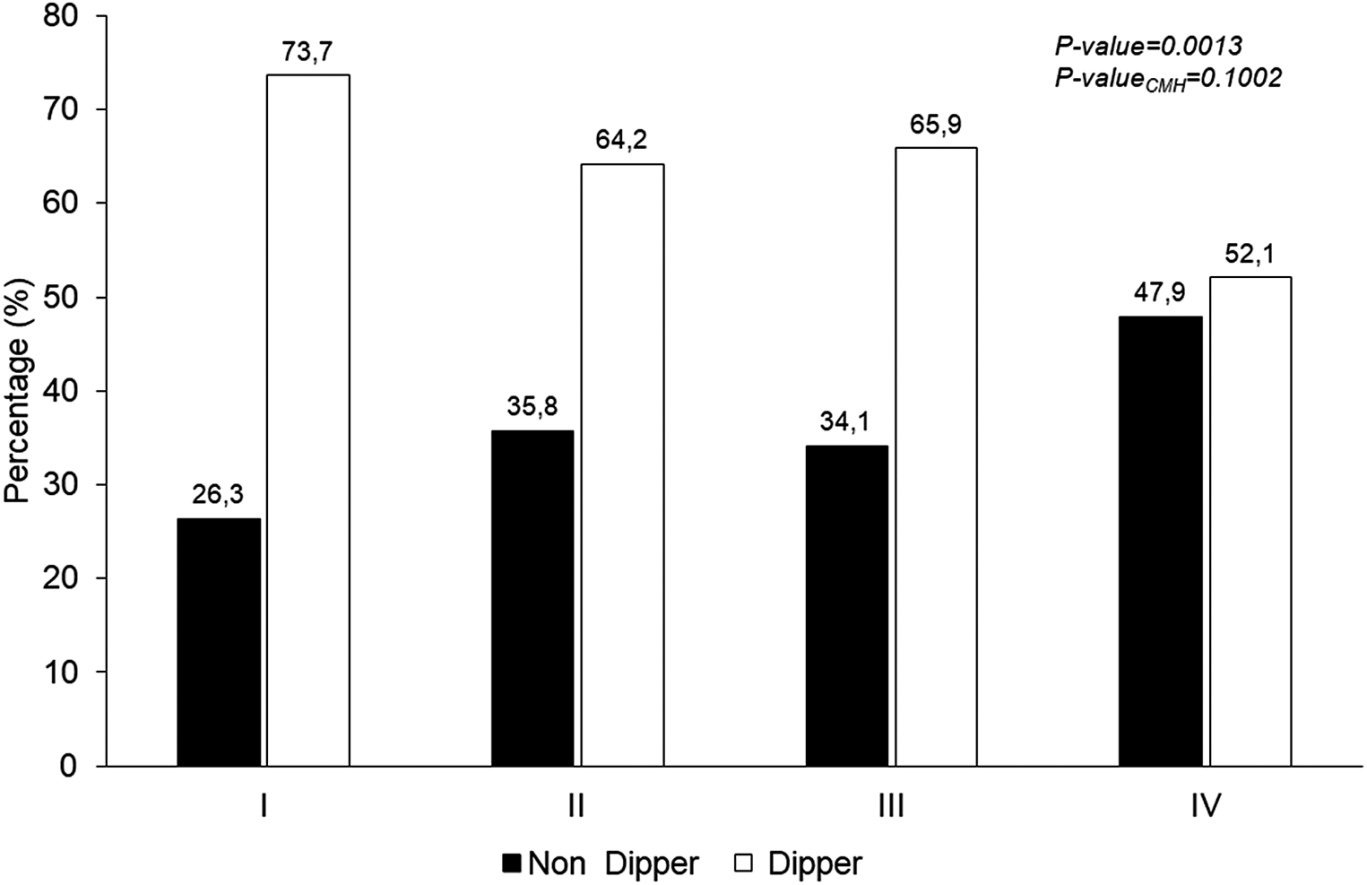
Prevalence rates of dipping/non-dipping pattern in the participants to the third survey of the PAMELA study stratified according to HMOD: (I) normal arterial stiffness, normal LVMI, and geometry; (II) normal arterial stiffness, LVH, or LV concentric remodeling; (III) increased arterial stiffness, normal LVMI, and geometry; and (IV) increased arterial stiffness, LVH, or LV concentric remodeling.

**Figure 4.**
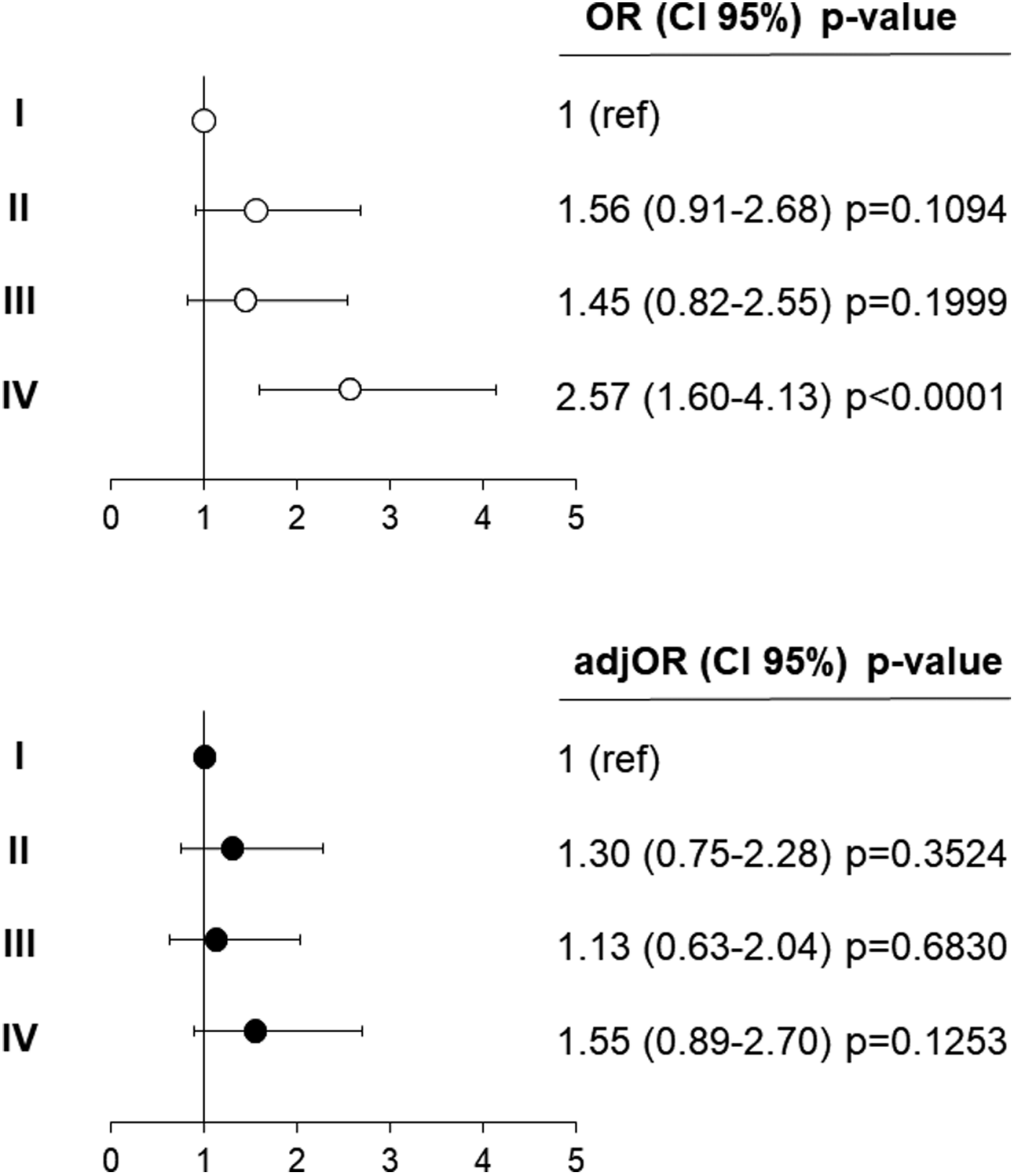
Odds Ratios (ORs), and 95% confidence interval, for the risk of non-dipping pattern in the participants to the third survey of the PAMELA study categorized according to HMOD (II = normal arterial stiffness, LV hypertrophy (LVH), or LV concentric remodeling; III = increased arterial stiffness, normal LVMI, and geometry; IV = increased arterial stiffness, LVH, or LV concentric remodeling) having as reference group I (normal arterial stiffness, normal LVMI, and geometry). The upper panel and bottom panels show unadjusted and sex/age-adjusted data, respectively. Adj, adjusted.

Figure 3 highlights the prevalence of dipping/ND patterns in the 4 groups categorized on the basis of target organ damage. The ND pattern ranged from 26.3% (group I) and 47.9% (group IV), with an intermediate and similar value in groups II and III. Demographic and clinical findings regarding the participants grouped according to dipping/ND pattern are reported in Supplementary Table 2. As reported in Figure 4, the association between combined cardiac and vascular organ damage (group IV) and ND pattern (OR = 2.57, CI: 1.60–4.13, *P* < 0.0001) disappeared after adjustment for age and sex (OR = 1.55, CI: 0.89–2.70, *P* = 0.125).

### Additional analyses

**Figure 5.**
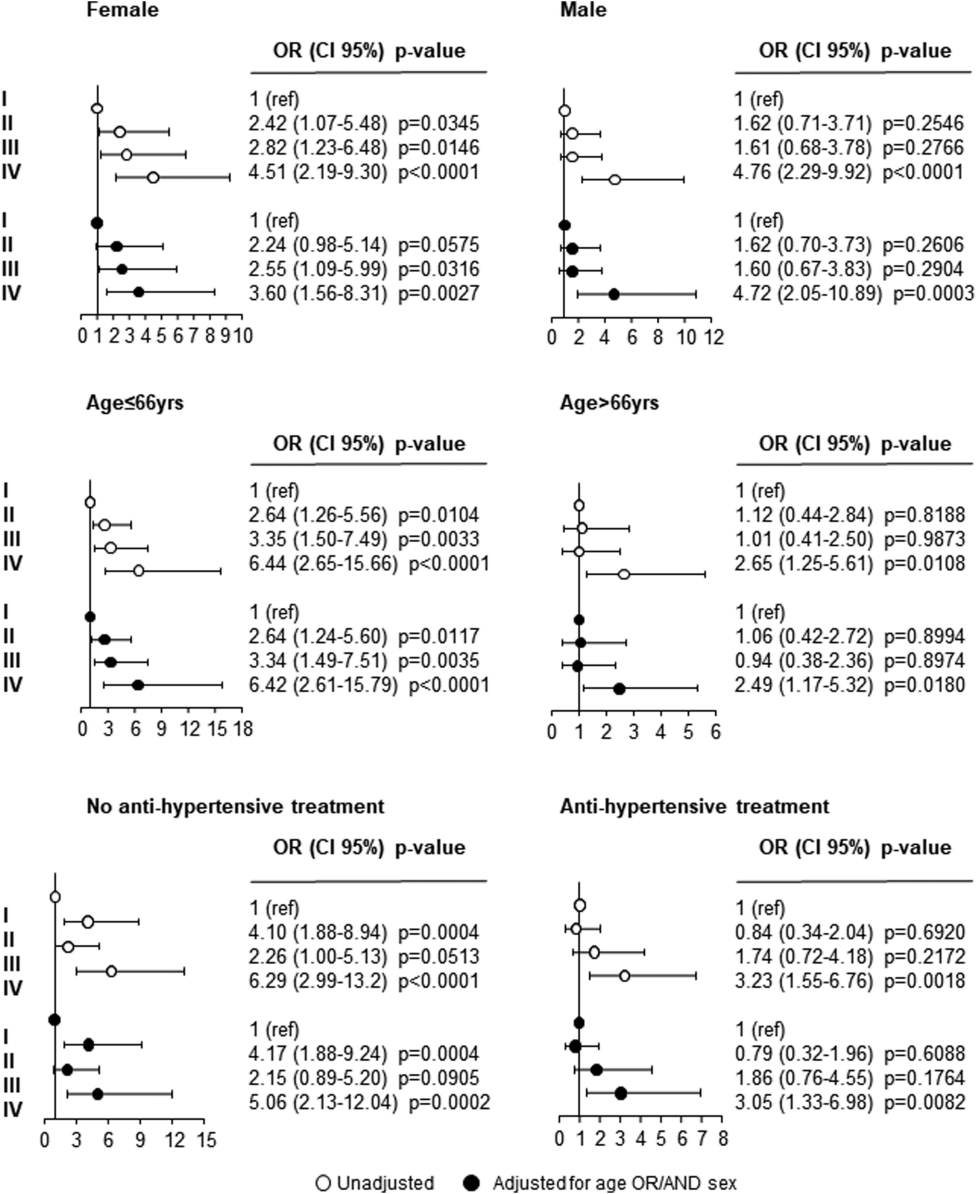
Odds Ratios (ORs), and 95% confidence interval, for the risk of sustained hypertension (SH) in the female (upper left panel), male (upper right panel), aged ≤66 years (middle left panel) >66 years (middle right panel), untreated (bottom left panel), and treated (bottom right panel) participants to the third survey of the PAMELA study categorized according to HMOD (II = normal arterial stiffness, LV hypertrophy (LVH), or LV concentric remodeling; III = increased arterial stiffness, normal LVMI, and geometry; IV = increased arterial stiffness, LVH, or LV concentric remodeling) having as reference group I (normal arterial stiffness, normal LVMI, and geometry). Unadjusted (white circles) and adjusted data (black circles).

Further analyses were performed for subgroups stratified by sex, age (<66 vs. ≥ 66 years), and antihypertensive treatment. Figure 5 illustrates ORs for SH into 3 groups with target organ damage separately in men and women and in participants aged under or over 66 years. The association between organ damage defined by increased arterial stiffness and LV remodeling or LVH (group IV) and the BP phenotype at higher cardiovascular risk, namely SH, persisted to be significant regardless of sex (OR = 3.60, CI: 1.56–8.31, *P* = 0.003 and 4.72, CI: 2.05–10.89, *P* = 0.0003, in women and men, respectively), age (OR = 6.42, CI: 2.61–15.79, *P* < 0.0001, and 2.49, CI: 1.17–5.32, *P* = 0.02, in participants aged under or over 66 years, respectively), and anti-hypertensive treatment (OR = 3.05, CI: 1.33–6.98, *P* = 0.008). This was not the case for the ND pattern. This association did not maintain its statistical significance in the separate adjusted analysis by sex, age, and anti-hypertensive treatment (Supplementary Figure 1).

## DISCUSSION

Arterial stiffness has progressively emerged in recent decades as a powerful indicator of CV disease and mortality risk thanks to a variety of non-invasive techniques providing a reliable metric of this index of subclinical artery damage. Despite that measurement of arterial stiffness has strong implications for refining CV risk stratification of individuals with hypertension, diabetes, dyslipidemia, and obesity it is still largely disregarded in clinical practice both in Europe and in the United States.^18^ The introduction of CAVI, based on the measurement of heart-ankle PWV, represents a significant step forward by correcting for the BP-dependency of inter-individual differences in PWV and offers many logistical and technical advantages that are beneficial for wider and more widespread clinical use.^19,20^ However, it remains to be clarified in which clinical conditions and settings the measurement of arterial stiffness with this method, simpler to implement than traditional metrics, can help the clinician in the management of patients at high CV risk.

The present study provides a new piece of information on the association between CAVI, LVM, and geometry, well-established markers of subclinical cardiac organ damage, with abnormal BP phenotypes, defined by combining office with ABP, and the magnitude of night-to-day BP fall (i.e., ND pattern) in elderly participants to the third PAMELA survey. Our main results can be discussed as follows. One, we analyzed a cohort of individuals belonging to the general population treated in approximately 50% of cases with antihypertensive drugs, in which only 30% had normal in- and out-office BP values. This means that the vast majority of participants had isolated or combined high in-office and out-office BP thus suggesting that BP control in the community is still far from optimal. Two, in parallel to this BP control trend, the prevalence of participants without vascular and cardiac organ damage was just over one-third, while the isolated increase in arterial stiffness, isolated LV remodeling/LVH, and the combined vascular and cardiac organ damage occurred in 17%, 19%, and 29% of participants, respectively. In phenotyping cardiac organ damage we followed the echocardiographic criteria recommended by the 2023 ESH guidelines, using indexation by BSA for LVM in relation to the limited prevalence of obesity in the PAMELA population.^6^ Increased arterial stiffness by CAVI, as we specified in the “Methods” section, was identified according to the cut-off of 9 m/s, in line with what was suggested a few years ago by the Japanese Society for Vascular Failure.^17^ This diagnostic threshold has been further validated in prospective outcome studies. The Advanced Approach to Arterial Stiffness (TRIPLE-A Stiffness), a European multicentre prospective longitudinal study aimed to determine associations of CAVI with CV morbimortality, showed that incidence rates of outcomes according to CAVI stratification were higher in the highest stratum (CAVI > 9 m/s) and in particular in older subjects (≥60 years).^20^ In this sub-group, the optimal CAVI threshold (adjusted for conventional risk factors) for prediction of incident CV morbimortality was 9.2 m/s. In light of the above, it is possible to make two considerations: the CAVI threshold of 9 m/s reliably identifies increased arterial stiffness independently of ethnicity and clinical setting; unlike what has been hypothesized by some authors CAVI maintains its predictive role even in the elderly population.^21^

Our study adds to previous evidence the notion that the integrated search for arterial damage by CAVI and LV remodeling/LVH by standard echocardiography allows for improved identification of individuals belonging to the general population at high CV risk due to a sustained elevation of BP both in and outside of the clinical environment. In fact, participants with both vascular and cardiac organ damage had a 4 times (OR = 4.31, *P* < 0.001) greater risk of SH compared to their counterparts without organ damage. Notably, the association between increased CAVI and LV remodeling/LVH conferred an incremental value in discriminating SH compared to both isolated organ damage phenotypes whose ORs reflected a lower likelihood of risk (OR = 1.92, *P* = 0.03 for increased CAVI and OR = 2.02, *P* = 0.02 for LV remodeling/LVH). The strength of the association of both vascular and cardiac damage persisted in the separate analysis by sex, age, and antihypertensive treatment, whereas isolated vascular damage was associated with SH only in the subgroup of participants aged <66 years and isolated cardiac organ damage in women and younger subjects. It is noteworthy that the parallel increase in arterial stiffness and subclinical cardiac damage was present in a large fraction of our cohort (approximately 30%) thus expanding previous cross-sectional and longitudinal evidence conducted in different settings.^22,23^ Yoshida *et al*.^23^ reported that the greater progression in arterial stiffness, assessed by CAVI, in 317 participants without CV disease at initial evaluation, was significantly associated with adverse LV remodeling during a median follow-up of 27 months.

A further result of our study deserves mention. We failed to find significant associations between elevated arterial stiffness, LV remodeling/LVH, and more importantly of the combination of these 2 markers of organ damage with ND pattern. The relationship between organ damage and alterations in the circadian rhythm of BP has been the subject of an impressive amount of studies over the last 40 years, with often inconsistent results. Without going into detail on this topic, many research groups, including ours, suggested that absolute nocturnal BP level rather than the magnitude of BP fall from daytime values is independently linked to subclinical organ damage.^24,25^

### Limitations

Some limitations of our study merit to be recognized. First, our findings refer to a selected cohort of elderly Southern European participants with a low prevalence of obesity and comorbidities, and thus, our data should not be extrapolated to populations with different ethnic and clinical characteristics. Second, non-attendance at follow-up of older or non-compliant participants and those who had CV events may have caused selection bias and consequently influenced the results. Third, a single ABPM was performed and the awake-asleep time was defined by wide fixed time periods. Both methodological aspects may have limitations because has been shown that the ND pattern has poor reproducibility over time and fixed time periods may not accurately reflect the actual night rest time.

Fourth, we limited our investigation to the BP phenotype (i.e., SH) characterized by a more unfavorable CV prognosis compared to WCH and MH. Fifth, participants taking BP-lowering drugs were not excluded from the present analysis in order to better reflect real-world conditions at the community level.

In conclusion, the present study highlights that the combined search for vascular and cardiac organ damage by CAVI and standard echocardiography has an incremental value in the identification of both in- and out-office hypertension, the BP phenotype with the highest CV risk. As a consequence, our findings suggest a more widespread implementation of the assessment of arterial stiffness in clinical practice to optimize CV risk stratification.

## Supplementary Material

hpae106_suppl_Supplementary_Table_S1

hpae106_suppl_Supplementary_Table_S2

hpae106_suppl_Supplementary_Figure

## Data Availability

Data to verify study outcomes are available on request to the corresponding author from qualified clinical researchers with approval from an Institutional Review Board.
