# Supplementary material for: Ambulatory Blood Pressure Phenotypes, Arterial Stiffness, and Cardiac Remodeling
Source: Am J Hypertens. 2024 Aug 8;37(12):978–86. doi: 10.1093/ajh/hpae106 (PMC11565190; doi:10.1093/ajh/hpae106)
Supplement: hpae106_suppl_Supplementary_Table_S1 [file hpae106_suppl_supplementary_table_s1.docx]

**Supplementary Table 1.** Demographic and clinical characteristics of the participants to the third survey of the PAMELA study categorized according to blood pressure phenotypes : I) sustained normotension (NT); II) white coat hypertension (WCH); III) masked hypertension (MH) ; IV sustained hypertension (SH).

|  | NT | WCH | MH | SH | p-trend |
| --- | --- | --- | --- | --- | --- |
| Number | 153 | 49 | 119 | 170 |  |
| Age (years) | 65.4±9°* | 69.3±8.1° | 62.3±9.7* | 68.4±9.2 | 0.0776 |
| Male (%) | 43.1 | 55.1 | 49.6 | 56.5 | 0.0273 |
| Body mass index (kg/m^2^) | 25.6±4.6* | 26±4.3 | 26.3±3.9 | 27.1±4 | 0.001 |
| Waist circumference (cm) | 88.4±14.4* | 90.3±13.6 | 91.2±11.8 | 94.2±12.4 | <.0001 |
| Office SBP (mmHg) | 122.7±11.5^°* | 148.2±9° | 127.5±9.8* | 151.9±13.3 | <.0001 |
| Office DBP (mmHg) | 77±6.2^°* | 86.7±7.4°* | 80.2±6.4* | 89.7±7.7 | <.0001 |
| Office HR (mmHg) | 69.1±9.3 | 70.9±11.4 | 70.2±9.3 | 71.2±10.8 | 0.0823 |
| Home SBP (mmHg) | 116.2±11.1^°* | 128.8±12.7* | 124.4±13.5* | 140.3±14 | <.0001 |
| Home DBP (mmHg) | 72.4±6.6^°* | 76.1±7.7* | 79±8.5* | 82.1±8.8 | <.0001 |
| Home HR (mmHg) | 70.7±10.1 | 70.6±11 | 71.9±9.5 | 69.8±8.7 | 0.6012 |
| 24-h SBP (mmHg) | 119.8±6.6^°* | 124.7±4.7°* | 137±8.5* | 144.5±11.2 | <.0001 |
| 24-h DBP (mmHg) | 71.5±4.6°* | 73.4±4.7°* | 81±5.4 | 82.1±7 | <.0001 |
| 24-h HR (mmHg) | 71.4±7.2 | 71.8±9.4 | 73.3±7.6 | 71.5±7.5 | 0.6251 |
| Day-time SBP (mmHg) | 124.7±8°* | 128.6±5.6°* | 142.8±9.2* | 149.6±11.4 | <.0001 |
| Day-time DBP (mmHg) | 75.1±5.5°* | 76.6±5.4°* | 85.3±6.2 | 85.6±7.6 | <.0001 |
| Day-time HR (mmHg) | 74.7±7.7 | 75±10.1 | 76.7±7.9 | 74.8±8 | 0.6738 |
| Night-time SBP (mmHg) | 107.5±8.4^°* | 114.1±8.7°* | 122.2±12.2* | 131±15.7 | <.0001 |
| Night-time DBP (mmHg) | 62.2±5.5°* | 64.5±5.5°* | 70.3±7.1* | 72.6±8.6 | <.0001 |
| Night-time HR (mmHg) | 62.6±8.1 | 63.1±9 | 64.6±8.4 | 62.9±7.4 | 0.5435 |
| Antihypertensive treat (%) | 46.4 | 61.2° | 36.1* | 54.1 | 0.5176 |
| Total cholesterol (mg/dl) | 199.2±38.6 | 193.7±36.8 | 205.2±33.7 | 201.6±37.7 | 0.3384 |
| HDL cholesterol (mg/dl) | 61.3±18.3* | 61.3±19.8 | 59.4±16.5 | 56±15.5 | 0.005 |
| Serum glucose (mg/dl) | 88 (82-95)^* | 94 (86-104) | 89 (85-100) | 92 (84-102) | 0.0291 |
| Triglycerides (mg/dl) | 89.5 (69-115) | 97 (74-121.5) | 99 (76-128) | 102.5 (76-133) | 0.0566 |
| Uric acid (mg/dl) | 4.9±1.31 | 5.05±1.4 | 5.14±1.16 | 5.05±1.24 | 0.2414 |
| Serum creatinine (mg/dl) | 0.92±0.24 | 0.96±0.33 | 0.92±0.19 | 0.94±0.22 | 0.5958 |
| LVM/BSA (g/m^2^) | 78.8±17.8^* | 90.1±18.3 | 82.5±18.6* | 92.3±21.8 | <.0001 |
| CAVI (m/sec) | 8.7±1.5* | 9.4±1.5 | 8.9±2.2* | 9.9±2.4 | <.0001 |

For abbreviations see preceding table.

^p<0.05 vs WCH; °p<0.05 vs MH; *p<0.05 vs SH.
