# Supplementary material for: Ambulatory Blood Pressure Phenotypes, Arterial Stiffness, and Cardiac Remodeling
Source: Am J Hypertens. 2024 Aug 8;37(12):978–86. doi: 10.1093/ajh/hpae106 (PMC11565190; doi:10.1093/ajh/hpae106)
Supplement: hpae106_suppl_Supplementary_Table_S2 [file hpae106_suppl_supplementary_table_s2.docx]

**Supplementary Table 2.** Demographic and clinical characteristics of the participants to the third survey of the PAMELA study according to dipping and non-dipping pattern.

|  | Non Dipper | Dipper | p-value* |
| --- | --- | --- | --- |
| Number | 176 | 315 |  |
| Age (years) | 68.8±9.5 | 64.6±9.1 | <.0001 |
| Male (%) | 55.7 | 47.6 | 0.0866 |
| Body mass index (kg/m2) | 27±4.4 | 26±4.1 | 0.0077 |
| Waist circumference (cm) | 93.7±12.5 | 90±13.5 | 0.0025 |
| Office SBP (mmHg) | 140.2±18.7 | 134.5±16.5 | 0.0004 |
| Office DBP (mmHg) | 83.1±9.7 | 83.1±8.3 | 0.9784 |
| Office HR (mmHg) | 69.7±10.8 | 70.6±9.6 | 0.3811 |
| Home SBP (mmHg) | 130.8±17.8 | 126±15.1 | 0.0031 |
| Home DBP (mmHg) | 77.5±9.5 | 77.8±8.6 | 0.7631 |
| Home HR (mmHg) | 68.9±9.8 | 71.6±9.3 | 0.0029 |
| 24-h SBP (mmHg) | 134.4±15.7 | 132.3±12.4 | 0.1295 |
| 24-h DBP (mmHg) | 76.9±8.2 | 78±7 | 0.1397 |
| 24-h HR (mmHg) | 70±7.9 | 73±7.3 | <.0001 |
| Day-time SBP (mmHg) | 136.2±15.8 | 139.2±13.3 | 0.0343 |
| Day-time DBP (mmHg) | 79±8.6 | 82.7±7.6 | <.0001 |
| Day-time HR (mmHg) | 73±8.2 | 76.5±7.8 | <.0001 |
| Night-time SBP (mmHg) | 129.7±16.4 | 114.3±12.1 | <.0001 |
| Night-time DBP (mmHg) | 71.8±8.6 | 65.8±7.5 | <.0001 |
| Night-time HR (mmHg) | 62.3±8.8 | 63.7±7.5 | 0.0707 |
| Antihypertensive treat (%) | 60.8 | 41 | <.0001 |
| Total cholesterol (mg/dl) | 196.1±38.8 | 203.6±35.7 | 0.0313 |
| HDL cholesterol (mg/dl) | 55.8±16 | 60.8±17.6 | 0.002 |
| Serum glucose (mg/dl) | 91 (84.5-103) | 89 (84-98) | 0.1079 |
| Triglycerides (mg/dl) | 102 (77.5-140.5) | 91 (70-123) | 0.0031 |
| Uric acid (mg/dl) | 5.13±1.32 | 4.96±1.23 | 0.1479 |
| Serum creatinine (mg/dl) | 0.99±0.27 | 0.9±0.2 | 0.0004 |
| LVM/BSA (g/m^2^) | 91.2±22.5 | 82.3±18.2 | <.0001 |
| CAVI (m/sec) | 9.6±2.2 | 9±2 | 0.0022 |

For abbreviations see preceding table. * p-value dipper vs non dipper.
