# Supplementary figures and images for: Ambulatory Blood Pressure Phenotypes, Arterial Stiffness, and Cardiac Remodeling
Source: Am J Hypertens. 2024 Aug 8;37(12):978–86. doi: 10.1093/ajh/hpae106 (PMC11565190; doi:10.1093/ajh/hpae106)

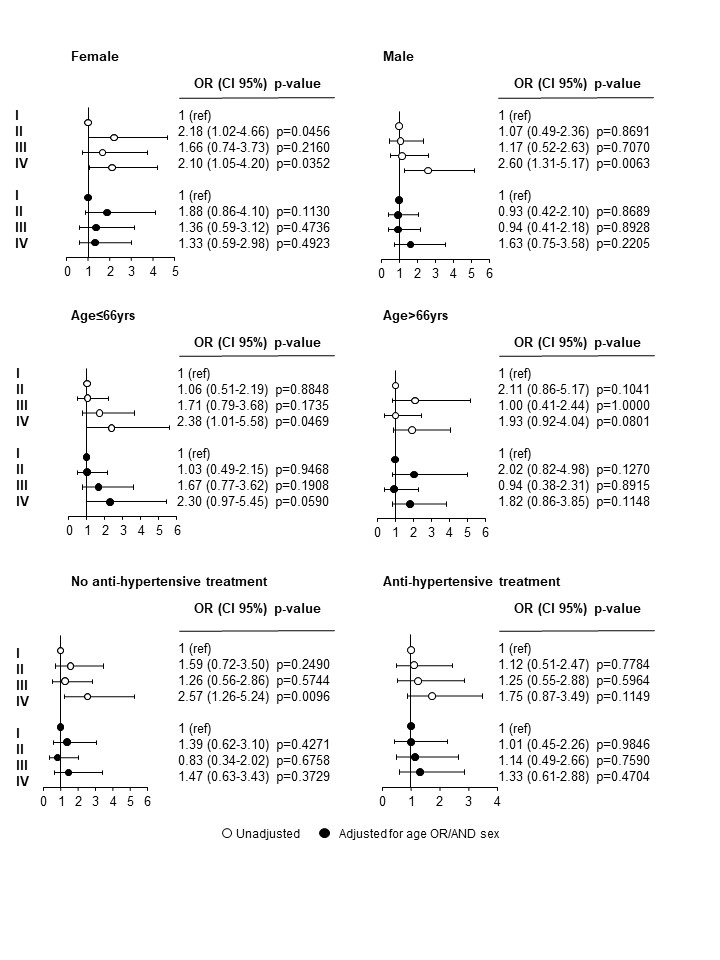

Supplement: hpae106_suppl_Supplementary_Figure [file hpae106_suppl_supplementary_figure.jpeg]
